# Relationship between the Size of the Samples and the Interpretation of the Mercury Intrusion Results of an Artificial Sandstone

**DOI:** 10.3390/ma11020201

**Published:** 2018-01-27

**Authors:** Hua Dong, Hongzhi Zhang, Yibing Zuo, Peng Gao, Guang Ye

**Affiliations:** 1 Microlab, Faculty of Civil Engineering and Geosciences, Delft University of Technology, Stevinweg 1, 2628 CN Delft, The Netherlands; H.Dong@tudelft.nl (H.D.); h.zhang-5@tudelft.nl (H.Z.); Y.Zuo@tudelft.nl (Y.Z.); P.Gao@tudelft.nl (P.G.); 2 School of Materials Science and Engineering, South China University of Technology, Guangzhou 510640, China

**Keywords:** artificial sandstone, pore structure, mercury intrusion, effect of sample size, simulation

## Abstract

Mercury intrusion porosimetry (MIP) measurements are widely used to determine pore throat size distribution (PSD) curves of porous materials. The pore throat size of porous materials has been used to estimate their compressive strength and air permeability. However, the effect of sample size on the determined PSD curves is often overlooked. In pursuit of a better understanding of the effect of sample size on mercury intrusion into porous materials, a combined experimental and numerical approach was applied. Quartz sand and epoxy resin were mixed to form artificial sandstone. Digital microstructures of the sandstone were obtained by using X-ray computed tomography (CT scan) technique. PSD curves of the artificial sandstone with different sample sizes were determined both by MIP measurement and by simulation of mercury intrusion (i.e., MIP simulation). Percolation analysis was performed on mercury-intruded pores in the digital microstructures. The PSD curves determined both by MIP measurements and by MIP simulations show that there was a significant effect of sample size on mercury intrusion before percolation of mercury-intruded pores. The effect of sample size decreased with the increasing pressure. After the mercury-intruded pores percolated through the samples, the effect of sample size on mercury intrusion became minor. The pore throat size of the artificial sandstone was used to estimate the air permeability using the relation proposed in the literature. The calculated air permeability of the smaller sandstone sample was higher. However, in principle, the air permeability of sandstone samples should be independent of the sample size. Two main conclusions can be drawn: (1) a fixed sample size should be used in MIP measurements or MIP simulation so that the PSD curves of different samples can be properly compared, (2) sample size needs to be considered when the pore throat size determined by MIP measurement is used for estimating air permeability.

## 1. Introduction

The pore structure of porous materials (e.g., rocks and cement-based materials) has an important role in mechanical and transport properties [1]. The pore structure of porous material can be characterized by a number of parameters, including porosity, hydraulic radius, specific surface area, threshold diameter, and pore size distribution [2]. Several methods can be used to characterize the pore structure of porous materials, such as gas adsorption (for pores smaller than about 10 nm in diameter), thermoporometry (for pores smaller than about 100 nm in diameter), nuclear magnetic resonance (for pores smaller than about 10 μm in diameter) and optical/electric microscopy [2]. Mercury intrusion porosimetry (MIP) is used to characterize the pore structure of porous materials for pore throat sizes from several nm to hundreds of μm [3,4,5]. The above techniques for characterizing pore structure have been comprehensively reviewed and compared in References [6,7,8]. The current study is limited to MIP technique for pore structure characterization. 

In MIP, the samples are placed in a pressure chamber and evacuated. Mercury is intruded into the samples under an increasing pressure. Pore throat size distribution (PSD) can be calculated according to the Washburn equation (Equation (1)), which relates the applied pressure P to the corresponding pore throat size $d_{c}$ [3].

(1) $$d_{c}=-4\sigma \cos\theta /P,$$

where σ is the surface tension of mercury and θ is the contact angle of mercury on the solid. 

Two assumptions have been made to convert the pressure P to the pore throat size $d_{c}$: (1) the pores are cylindrical, and (2) the pores are entirely and equally accessible to the outer surface of the specimen. However, most porous materials cannot fulfil these two assumptions. MIP measurements are believed to systematically misallocate the sizes of almost all pores in porous materials, and assign to sizes smaller than the actual ones due to the presence of “throat” and “ink bottle” pores [9]. In spite of the above-mentioned drawbacks of MIP measurements, efforts have been made to relate the pore throat size as determined by MIP measurements to the compressive strength of cement pastes [10,11] and the air permeability of rocks [12,13,14,15]. Odler and Rößler (1985) [10] related the compressive strength of cement paste to the volume fraction of pores (for pores with d_c_ < 10 nm, 10 nm ≤ d_c_ ≤ 100 nm, and d_c_ > 100 nm, respectively). The volume fraction of pores was calculated from the PSD curves. Katz and Thompson (1986) [12] described the permeability as a function of the characteristic length, the conductivity of a rock saturated with a brine solution, and the conductivity of the solution. The characteristic length refers to the pore throat size of the rock when the mercury-intruded pores start to percolate through the rock. Stanley (1980) [13], Pittman (1992) [14], and Rezaee et al. (2006) [15] related the permeability of rock to the porosity and the pore throat size that corresponded to a certain saturation of mercury (e.g., from 5 to 75%). 

It has been reported that the pore throat size determined by MIP measurements are influenced by many factors, such as contact angle between mercury and solids in porous material [16], damage of samples [5] that is possibly caused by crushing [17], sample drying before MIP measurement [18], or pressurized mercury during MIP measurement [19]. Sample size was also found to have an effect on PSD curves determined by MIP measurements [17,20,21,22,23]. Instructions of most laboratory-grade mercury intrusion porosimeters (e.g., AutoPore V Series and Micromeritics 9405 CE Auto Pore III) only specify the maximum sample size (e.g., diameter = 2.5 mm, height = 2.5 cm). The actual sample size is decided by the users.

Investigation of the effect of sample size on the PSD curves determined by mercury intrusion is important, because it provides a basis for a proper application of the PSD curves, such as the estimation of the compressive strength or the air permeability of materials. Bager et al. (1975) [20] conducted MIP experiments on hardened cement pastes with a water-to-cement (w/c) ratio of 0.4. They found that the PSD showed an increasing fraction of large pores with a decreasing sample size (0.149–2 mm). Hearn et al. (1992) also reported that the PSD curves of cement pastes (water-to-cement ratio = 0.5, water cured, 2-year old) differed with the dimensions [17]. Larson et al. (1981) [21] applied percolation theory to investigate the effect of sample size on the determined PSD by the drainage curve of wetting phase in porous materials. They stated that samples with a smaller size would increase the accessibility of pore space. On the contrary, Moro et al. (2002) [22] found that the accessibility of the pores to mercury increased as the sample size increased from 1 to 20 mm. The increased accessibility was explained by the damage due to sample drying before MIP measurements. They stated that sample drying might cause less damage to small samples than to large samples. Moreover, Ma (2014) [23] reported an insignificant effect of the sample size of cement pastes when the sample size was smaller than 5 mm, and thus recommended that the maximum sample size of 5 mm should be used to reduce the sample size effect. 

As stated above, the effect of sample size on mercury intrusion in MIP measurements is determined by combining factors (e.g., accessibility of mercury into samples and damage due to drying of samples). A better insight into the effect of sample size requires the understanding of each single factor. However, it is difficult to separate these two factors in experiments. Simulation of mercury intrusion (MIP simulation) enables a single-parameter study on the effect of sample size, in terms of accessibility of mercury into the samples [24,25,26]. Garboczi et al. (1991) [24] stated that there was no significant effect of sample size in MIP simulation. Munch et al. (2008) [25] and Huy Do et al. (2013) [26] simulated mercury intrusion into digital microstructures of cement pastes and determined the PSD curves. They found that large samples exhibited finer pore structures than small samples by comparing the PSD curves. However, detailed discussion of the effect of sample size on the determined PSD curves is still lacking. 

The main aim of this study is to experimentally and numerically investigate the relationship between the sample size and the PSD curves determined by using MIP techniques. Artificial sandstone samples with different bulk sizes ($10\times 10\times 10\;{\mathrm{mm}}^{3}$ and $5\times 5\times 5\;{\mathrm{mm}}^{3}$) were prepared by mixing quartz sand and epoxy resin. PSD curves of sandstone sample with different sizes were determined by MIP measurements. The digital microstructures of artificial sandstone were reconstructed by using X-ray computed tomography (CT scan) technique. 

Using an artificial sandstone can be especially advantageous. The pore structure of the artificial sandstone can be tailored so its microstructure can be properly reconstructed by CT. However, it is challenging to properly obtain the digital microstructure of natural sandstone, because the pore throat size of natural sandstone may range from nm to μm [27], and the spatial resolution of CT is limited. For instance, Cnudde et al. (2011) [28] used a micro-CT to obtain the digital microstructure of Bray sandstone, which had a minimum pore throat size of 4.7 μm (determined by MIP measurement). Scanning of the Bray sandstone with a cross-section of 6.6 × 7.4 mm^2^ resulted in a resolution of a micro-CT data of 7.4 μm [28]. The spatial resolution could be increased to 0.5 μm/voxel at the cost of a reduced size of the cross-section [29]. 

The artificial sandstone prepared in this study had a coarse pore structure with a minimum pore throat size of about 10 μm (determined by MIP measurement). The digital microstructure of the artificial sandstone (size: $10\times 10\times 10\;{\mathrm{mm}}^{3}$) could therefore be properly reconstructed by using micro-CT in laboratory. Simulation of mercury intrusion was performed on the digital microstructures with different sizes (e.g., $10\times 10\times 10\;{\mathrm{mm}}^{3}$, $5\times 5\times 5\;{\mathrm{mm}}^{3}$, $3.33\times 3.33\times 3.33\;{\mathrm{mm}}^{3},$ and $2.5\times 2.5\times 2.5\;{\mathrm{mm}}^{3}$). PSD curves of the digital microstructures were determined, followed by a percolation analysis on the mercury-intruded pores. 

Considering that the MIP technique has been widely adopted to characterize the pore structure of natural and artificial porous media, implications regarding the sample size are given to the pore structure characterization of these materials using MIP technique. The pore throat size of rocks determined by MIP measurements has been used to estimate the air permeability of the rocks [12,14,15]. However, the sample size used in the MIP measurements for determination of the pore throat size was often not mentioned. In this study, air permeability of the artificial sandstone with different sizes was estimated based on the pore throat size. The effect of sample size on the estimated air permeability of artificial sandstone was evaluated. Implications are given for a proper use of the pore throat size in the estimation of air permeability of rocks. 

## 2. Sample Preparation and Derivation of Digital Microstructures

### 2.1. Materials and Sample Preparation

**Figure 1:**
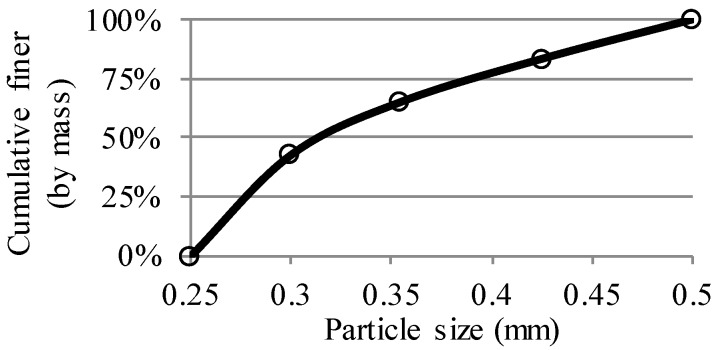
Particle size distribution of the quartz sand used for the preparation of artificial sandstone, as determined by sieving.

Artificial sandstone with a coarse pore structure was fabricated by mixing natural quartz sand with epoxy resin. The mass ratio of sand to epoxy resin was 1:0.26. Description of the raw materials is listed as below:

- Quartz sand (particle size: 0.25–0.5 mm): the particle distribution is shown in Figure 1.
- Epoxy resin: the epoxy resin consists of Conpox Harpiks BY 158 and Haerder HY 2996. The mass ratio of hardener to epoxy resin was 0.3. The elastic modulus of the epoxy resin after complete polymerization was 3.8 GPa. The deformation of the epoxy resin under the pressure of mercury in MIP measurements was considered to be minor.

**Figure 2:**
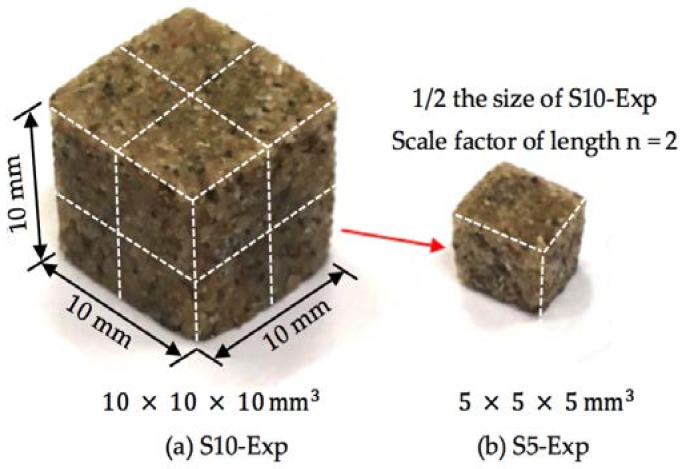
Sandstone samples with different sizes for MIP measurements. The large sample (S10-Exp) is taken as a reference. The length of the small samples (S5-Exp) is scaled down by a factor *n* = 2.

After mixing, the mixture was cast in two different moulds (dimensions: diameter = 15 mm, height = 100 mm, and 40 mm × 40 mm×40 mm). The sandstone specimens were demoulded one day after casting (at one day, the epoxy resin had hardened and obtained sufficient strength). Small samples were obtained by cutting with a 1 mm-thick diamond blade followed by grinding on sandpaper with a grit size P320. The small samples were used for different tests, including MIP measurement (denoted as S10-Exp and S5-Exp, shown in Figure 2), CT scan test, and compressive strength test (denoted as C15) (see Table 1). The numbers of samples with different sizes are presented in Table 1. It has to be pointed out that the numbers of samples for studying the effect of sample size (mostly on mechanical properties) vary in different experiments (e.g., from 1 to 10), and the standard deviation of experimental results have often been presented [30,31,32]. In this study, the total volume of the samples with a certain size was kept constant (i.e., 10 mm^3^). Duplicate samples of S10-Exp were prepared to test the repeatability of the MIP measurements on artificial sandstone samples.

### 2.2. Derivation of Digital Microstructures of the Sandstone–CT Scan

**Figure 3:**
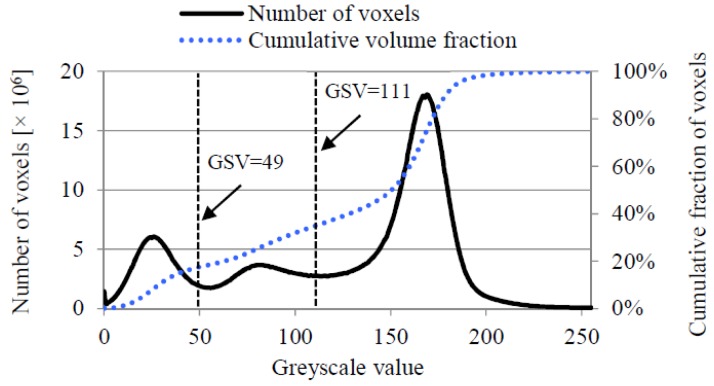
Greyscale value histogram and cumulative fraction of the voxels of the artificial sandstone.

**Figure 4:**
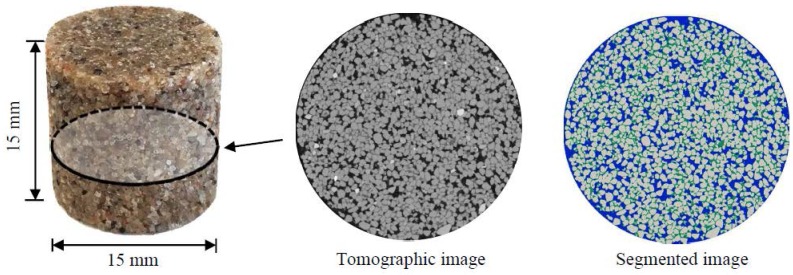
Sandstone sample prepared for CT scan test. The sandstone was made of quartz sand and epoxy resin, and had a bulk density of 2130 kg/m^3^. In the segmented image, quartz sand is shown in grey, epoxy resin in green, and pores in blue.

**Figure 5:**
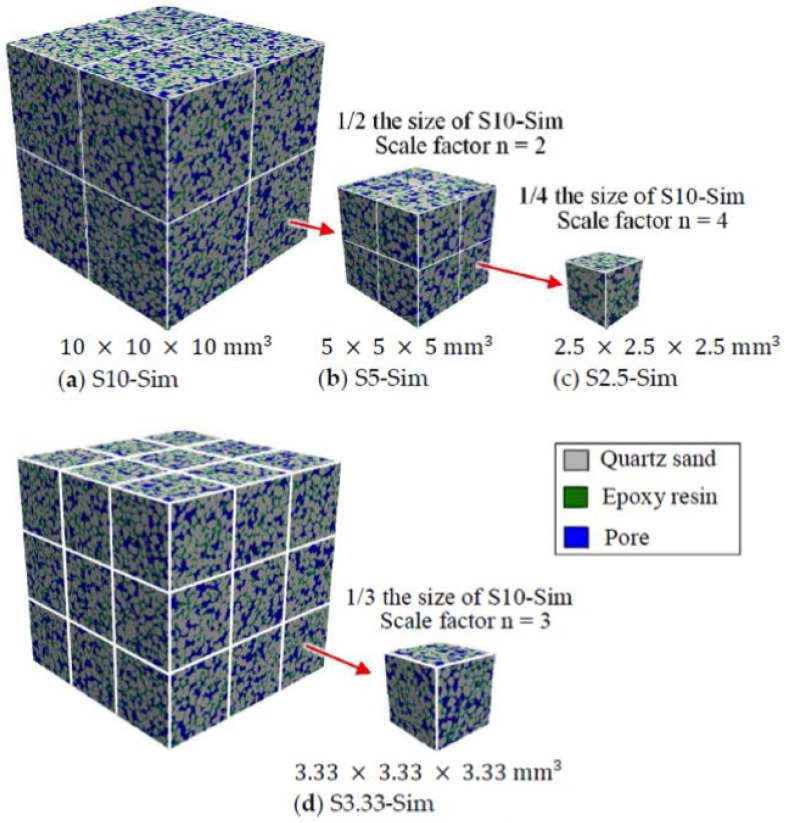
Digital microstructure of sandstone samples with different sizes (digital resolution = 10 μm/voxel). Quartz sand is shown in grey, epoxy resin in green, and pores in blue. The size of the large sample (S10-Sim) is taken as a reference. The lengths of small samples (i.e., S5-Sim, S3.33-Sim, and S2.5-Sim) are scaled down by a factor *n* = 2, 3, and 4, respectively.

The digital microstructure of the sandstone (C15) was obtained by using a Micro CT-Scanner (Phoenix Nanotom, Boston, MA, USA). The original image data was transformed to 8-bit greyscale data, which was considered sufficient for segmentation of different phases (sands, epoxy, and pores). Figure 3 shows the greyscale value (GSV) histogram and the cumulative fraction of the voxels. A global threshold approach was used to identify different phases [33]. Given a specified threshold of GSV (corresponding to the valley of the histogram curve of voxels), the histogram curve is divided into continuous regions. Voxels are identified as pores when 0≤GSV≤49, epoxy resin when 49<GSV≤111, and quartz sand when 111<GSV≤255 (Figure 3). The volume fraction of pores, epoxy resin, and quartz sand in the bulk sample is 17.4%, 18.6%, and 64%, respectively. The connectivity of the pores is 93.7% (voxels are considered connected when sharing a face). Figure 4 shows sandstone (C15) for the CT scan test and a two-dimensional CT scan image before and after segmentation. A volume of interest (VOI) with 1000×1000×1000 voxels^3^ (digital resolution = 10 μm/voxel) was taken from the central part of the sample (C15) for pore structure characterization. Figure 5 shows digital microstructures of sandstone samples with different sizes (i.e., S10-Sim, S5-Sim and S3.33-Sim S2.5-Sim, listed in Table 1). 

**Figure 6:**
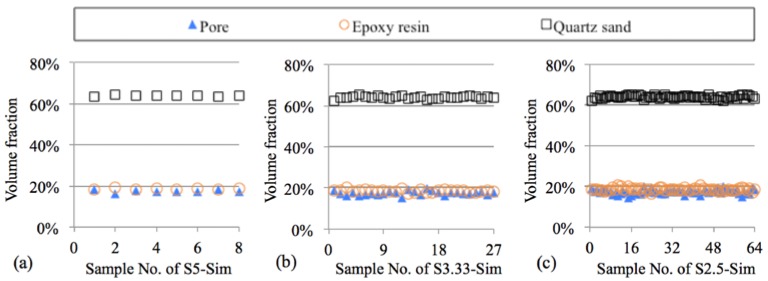
Volume fraction of pore, epoxy resin, and quartz sand in digital microstructures of sandstone samples with different sizes. (**a**) S5-Sim (8 samples), (**b**) S3.33-Sim (27 samples), (**c**) S2.5-Sim (64 samples).

Detailed description of the digital samples (S10-Sim, S5-Sim, S3.33-Sim, and S2.5-Sim) used for MIP simulation is listed in Table 2. The volume fractions of pore, epoxy resin, and quartz sand of the digital samples are presented in Figure 6. The variations of the volume fraction of each phase in the digital samples (S5-Sim, S3.33-Sim, and S2.5-Sim) are small, indicating a relatively homogeneous microstructure of the artificial sandstone. 

## 3. Methods for Determining PSD Curves

### 3.1. MIP Measurements

Before MIP measurement, the sandstone samples were dried in the oven at 40 °C for 2 days for a fast removal of moisture in the samples, and stored in a vacuum chamber until the weight loss was less than 0.01% by mass of the sample per day. MIP measurements were carried out by using a Micrometrics PoroSizer^®^ 9320. The contact angle and surface tension of mercury were taken as 141° and 485 nN/m, respectively [34]. In view of the coarse pore structure of artificial sandstone, the MIP measurements were performed at low pressures (0.0037–0.14 MPa). The corresponding pore throat size ranged from 404 to 10.8 μm. Note that the compression tests of the artificial sandstone were performed to ensure that the artificial sandstone could endure the pressure imposed for mercury intrusion during MIP measurements. The compressive strength of the artificial sandstone was 5.02±0.07 MPa (average value of three specimens with the same size as C15 listed in Table 1), which is much higher than the maximum pressure of mercury (i.e., 0.14 MPa). The shape and the size of samples may have an effect on the tested compressive strength [32], but are beyond the scope of this study.

### 3.2. Simulation of Mercury Intrusion

**Figure 7:**
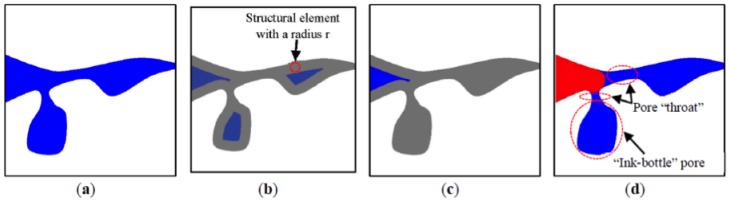
Two-dimensional illustration of steps for simulating mercury intrusion into porous material; solid is shown in white, pore in blue, and mercury in red. (**a**) original porous system, (**b**) pore space after erosion by a structural element, (**c**) pore space connected to the surfaces of material, (**d**) pore space after dilation; area in red represents mercury-intruded pore space.

**Figure 8:**
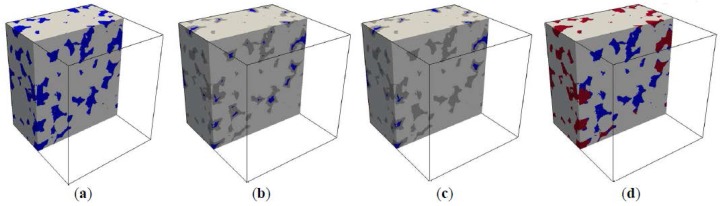
Illustration of steps for simulating mercury intrusion into digital microstructure of S2.5-Sim (d_c_ = 120 μm), solid is shown in light grey, pore in blue, and mercury in red. (**a**) original porous system, (**b**) pore space after erosion by a structural element, (**c**) pore space connected to the surfaces of the material, (**d**) pore space after dilation; area in red represents mercury-intruded pore space.

Mercury intrusion into digital microstructure is simulated based on image analysis of pore morphology, following the methodology adopted earlier by Hazlett et al. (1995) [35]. A detailed description of this methodology can also be found in Vogel et al. (2005) [36]. In the MIP simulation, pressure does not explicitly come into the algorithm. Instead, the corresponding pore throat size $d_{c}$ (refer to the Washburn equation) is directly used. Figure 7a and Figure 8a show a schematic pore structure and the digital microstructure of S5-Sim, respectively. Mercury intrusion into the pore structures is simulated by performing three main steps:

1. The pore space is eroded by circles/spheres (also called structuring element) with a radius r. The erosion process takes place incrementally from the pore surface to its medial axis, and removes voxels from the pore space. The eroded pore pixels/voxels are shown in grey (Figure 7b and Figure 8b).
2. Connectivity of the eroded pore space from the intruding surfaces is checked. Note that pixels/voxels are considered as connected when sharing an edge/face. Mercury is considered accessible to the pores when the eroded pore space has a continuous connection to the surfaces of the sample. Hence, all pores separated from intruding surfaces are removed from the eroded pore space (Figure 7c and Figure 8c).
3. To determine mercury-intruded pore space, the eroded set is dilated using the circles/spheres with the same radius r as for erosion (Figure 7d and Figure 8d). This finally leads to the opening of the pore space. The corresponding pore throat size is therefore $d_{c}=2r$.

Simulation is continued consecutively with smaller spheres by repeating these three steps. Based on the volume of intruded mercury with a certain structuring element, the PSD curve of the digital microstructure is determined.

## 4. Results and Discussion

### 4.1. PSD Curves Determined by MIP Measurements

**Figure 9:**
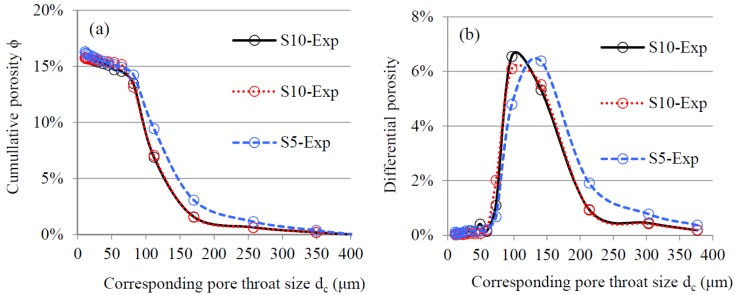
(**a**) Cumulative porosity and (**b**) differential porosity of artificial sandstone samples with different sizes as determined by laboratory MIP measurements.

Figure 9 presents the PSD curves of the samples with different sizes (S10-Exp and S5-Exp) determined by MIP. Duplicate MIP measurements were conducted on the large sample (S10-Exp), with one sample in each measurement. The two cumulative porosity curves of S10-Exp (black solid and red dotted lines in Figure 9a) show that the repeatability of the measurement is satisfactory. In order to keep the same volume of samples in each MIP measurement, eight small samples (S5-Exp) were used in a batch when performing MIP measurements. The increase of cumulative porosity of both large and small samples was insignificant when the pore throat size was smaller than 50 μm. This means that most pores in the artificial sandstone were larger than 50 μm. A clear difference in the PSD curves could be observed between the large sample (S10-Exp) and the small samples (S5-Exp). Note that the difference in the PSD curves between large (S10-Exp) and the batch of small samples (S5-Exp) is not caused by the variability of MIP measurements, indicated by the superimposing PSD curves of large samples. When $d_{c}\geq 80\;\mu m$, the cumulative porosity of large sample was lower than that of small samples (S10-Exp), indicating a significant effect of sample size on the PSD curves. When $d_{c}<80\;\mu m$, the effect of sample size was minor. The experimental results are in accordance with the finding of Bager et al. (1975) [20]. 

To further evaluate the effect of sample size on mercury intrusion, differential porosity $\phi_{d}$ was introduced. The differential porosity is the volume of pores ascribed to a certain pore throat size in a unit volume of porous material. $\phi_{d}$ is calculated based on the cumulative porosity curve:

(2) $$\phi_{d}=∆\phi /∆d_{c}.$$

Figure 9b presents the differential porosity of the sandstone samples (S10-Exp and S15-Exp). The curve of small samples (S5-Sim) showed higher differential porosity than large samples (S10-Exp) when d_c_ ≥120 μm, and lower differential porosity when $d_{c}$ <120 μm. The cumulative porosity of S10-Exp and S15-Exp at $d_{c}$ = 120 μm was 5.8% and 8.3%, respectively. The difference in the differential porosity of samples with different sizes was also reported by Hearn et al. (1992) [17].

### 4.2. Simulation of Mercury Intrusion

#### 4.2.1. PSD Curves Determined by MIP Simulation

**Figure 10:**
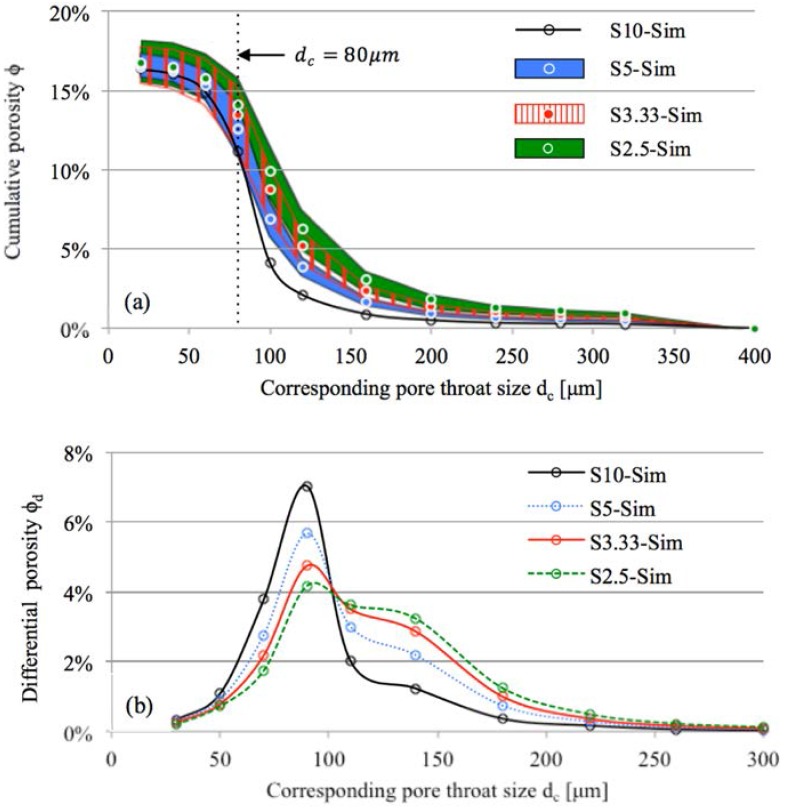
Pore throat size of artificial sandstone samples with different sizes determined by MIP simulation. (**a**) Cumulative porosity. The cumulative porosity curves of smaller samples (S5-Sim, S3.33-Sim and S2.5-Sim) are shown in shaded area, indicating standard deviation of the simulated results. (**b**) Differential porosity. The differential porosity curves of the small samples are calculated from the average values of the cumulative porosity.

By using the methodology presented in Section 3.2, the cumulative porosity of digital microstructures of sandstone samples was determined by MIP simulation. Figure 10a presents the PSD curves of the sandstone samples with different sizes (i.e., S10-Sim, S5-Sim, S3.33-Sim, and S2.5-Sim). The cumulative porosity was noticeably higher for smaller samples than for larger samples when $d_{c}\geq 80\;\mu m$. The significant difference in the cumulative porosity curves is in accordance with the experimental results shown in Figure 9a. The difference is also in agreement with the findings reported by Huy Do et al. (2013) [26]. However, Garboczi et al. (1991) [24] reported insignificant difference in cumulative porosity curves for two-dimensional model porous materials with different sizes (the model porous materials are represented by a set of digitized solid circles). 

Note that the cumulative porosity at a certain $d_{c}$ determined by MIP simulation (Figure 10a) was lower than that determined by MIP measurements (Figure 9a). This can be explained by the use of a spherical structuring element in the MIP simulation, which is placed in the pore space to determine the mercury–air interface. The spherical structuring element had two identical principle curvatures (i.e., the reciprocal of radius r). However, in reality (MIP measurements) there may exist two different curvatures at the mercury–air interface [37]. Using a spherical structuring element underestimates the cumulative porosity in MIP simulation, but will not impair the evaluation of the effect of sample size on mercury intrusion. Detailed investigation of the structuring element for MIP simulation is out of the scope of this paper.

Figure 10b presents the differential porosity curve $\phi_{d}$ of the sandstone samples as determined by MIP simulation. Samples with smaller size exhibit higher differential porosity when $d_{c}>100\;\mu m$ and lower differential porosity when $d_{c}<100\;\mu m$. The difference can be quantitatively evaluated from the cumulative porosity curves. When d_c_ = 100 μm, the cumulative porosity of S10-Sim, S5-Sim, S3.33-Sim, and S2.5-Sim was 4.12%, 6.87%, 9.89%, and 14.06%, respectively (see Figure 10a). Taking S10-Sim as a reference, the volume fraction of pores larger than 100 μm in small samples (i.e., S5-Sim, S3.33-Sim, and S2.5-Sim) was overestimated, while the volume fraction of pores smaller than 100 μm in small samples was underestimated. 

#### 4.2.2. Percolation Analysis of Mercury-Intruded Pores

**Figure 11:**
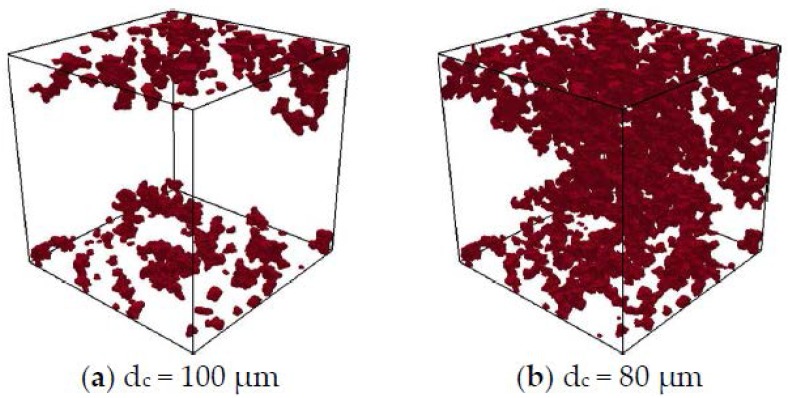
Percolation analysis of mercury-intruded pores in sandstone sample (S5-Sim). Mercury (in red) is intruded from the top and bottom surfaces of the sample. (**a**) The mercury has only penetrated the outer part (adjacent to the top and bottom surfaces) of the sample when d_c_ = 100 μm. (**b**) The mercury-intruded pores have percolated in the sample when d_c_ = 80 μm.

Percolation analysis of mercury-intruded pores was performed to investigate the accessibility of mercury to the pore space of sandstone samples. The mercury was intruded from two opposite surfaces of the digital microstructures at a certain $d_{c}$. Mercury-intruded pores were considered percolated as long as the mercury intruding from the top surface connected to the bottom surface of the sample. The pore throat size at which mercury-intruded pores get percolated is defined as the breakthrough pore throat size ($d_{\mathrm{perc}}$). According to Huy Do et al. (2013) [26], the breakthrough pore throat size is independent of sample size. Figure 11 illustrates the mercury-intruded pores in S5-Sim. Note that the percolation analysis of mercury-intruded pores gives the same value of $d_{\mathrm{perc}}$ when mercury is intruded from any pair of opposite surfaces of the sample (i.e., top and bottom, front and back, left and right surfaces of S5-Sim). When $d_{c}=100\;\mu m$, the mercury only reaches the outer part of the sample. When $d_{c}=80\;\mu m$, the mercury-intruded pores get percolated through the sample. Therefore, the breakthrough pore throat size falls between 80 μm to 100 μm. Before percolation ($d_{c}\geq 100\;\mu m$), the mercury can only penetrate to a certain depth of the sample. The depth is determined by the pore structure of the sample and is independent of the sample size. The volume fraction of mercury-intruded pores in the total volume of pores is lower for larger samples (see Figure 10a). After percolation ($d_{c}\leq 80\;\mu m$), the mercury penetrates through the samples, and the effect of sample size on mercury intrusion diminishes (see Figure 10a).

#### 4.2.3. Evaluation of the Effect of Sample Size

As shown in Figure 10a, the cumulative porosity of the artificial sandstone determined by mercury intrusion depends on sample size. To further evaluate the effect of sample size, the large sample (i.e., S10-Sim) is taken as a reference, and a ratio $R_{\phi }$ is defined as:

(3) $$R_{\phi }\left(d_{c}\right)=\frac{\phi_{\mathrm{Small}}\left(d_{c}\right)}{\phi_{S10-\mathrm{Sim}}\left(d_{c}\right)},$$

where $\phi_{\mathrm{Small}}\left(d_{c}\right)$ and $\phi_{S10-\mathrm{Sim}}\left(d_{c}\right)$ are the cumulative porosities of small sample (i.e., S5-Sim, S3.33-Sim, or S2.5-Sim) and large sample (i.e., S10-Sim) at pore throat size $d_{c}$, respectively. 

The value of $R_{\phi }\left(d_{c}\right)$ shows the extent of the discrepancy of the cumulative porosity curves between samples with different sizes. A higher value of $R_{\phi }$ indicates a greater effect of sample size on mercury intrusion. When the value is close to 1, the effect of sample size is considered minor. 

**Figure 12:**
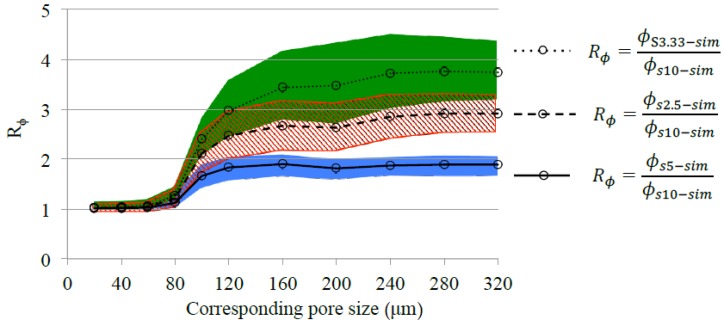
Effect of sample size on the cumulative porosity of mercury-intruded pores. $R_{s}$ is the ratio of the cumulative porosity of small sample (S5-Sim, S3.33-Sim, or S2.5-Sim) to that of large sample (S10-Sim). The shaded areas indicate standard deviations of the simulated results.

The ratio $R_{\phi }$ for different samples is plotted against the corresponding pore throat size $d_{c}$ in Figure 12. The evolution of $R_{\phi }$ can be divided into three stages. Discussion is made in the order of increasing pressure (or decreasing $d_{c}$):

1. $160\;\mu m\leq d_{c}\leq 320\;\mu m$: Mercury first intruded under a low pressure (e.g., corresponding pore throat size $d_{c}=320\;\mu m$), only vicinities of sample surfaces were filled with mercury. The ratio $R_{\phi }$ (i.e., $\phi_{s2.5-\mathrm{sim}}$/$\phi_{s10-\mathrm{sim}}$ and $\phi_{s5-\mathrm{sim}}$/$\phi_{s10-\mathrm{sim}}$) exhibited a value much greater than 1, indicating a significant effect of sample size on mercury intrusion. In this stage, for a small sample that is 1/n the size of the large sample (e.g., S10-Sim), the cumulative porosity of the small sample was scaled up by a factor of approximately n (e.g., $R_{\phi }=\phi_{s2.5-\mathrm{sim}}$/$\phi_{s10-\mathrm{sim}}\approx 4$, $R_{\phi }=\phi_{s3.33-\mathrm{sim}}$/$\phi_{s10-\mathrm{sim}}\approx 3$, and $R_{\phi }=\phi_{s5-\mathrm{sim}}$/$\phi_{s10-\mathrm{sim}}\approx 2$ when $d_{c}=320\;\mu m$).
2. $80\;\mu m\leq d_{c}<160\;\mu m$: As the pressure increased (e.g., $d_{c}$ decreases from 160 μm to 80 μm), mercury continued to penetrate into the samples. The value of $R_{\phi }$ decreased with the increasing pressure (or decreasing pore throat size $d_{c}$), but remained far greater than 1. The mercury-filled pores had percolated when the corresponding pore throat size $d_{c}=80\;\mu m$. This led to a smaller value of $R_{\phi }$ (approaching 1) for $\phi_{s2.5-\mathrm{sim}}$/$\phi_{s10-\mathrm{sim}}$, $\phi_{s3.33-\mathrm{sim}}$/$\phi_{s10-\mathrm{sim}}$, and $\phi_{s5-\mathrm{sim}}$/$\phi_{s10-\mathrm{sim}}$, indicating a minor effect of sample size on mercury intrusion under the pressure corresponding to $d_{c}=80\;\mu m$.
3. $d_{c}<80\;\mu m$: After the mercury-filled pores had percolated, the sample size had a negligible effect on mercury intrusion, as indicated by $R_{\phi }\approx 1$ for $\phi_{s2.5-\mathrm{sim}}$/$\phi_{s10-\mathrm{sim}}$, $\phi_{s3.33-\mathrm{sim}}$/$\phi_{s10-\mathrm{sim}}$ and $\phi_{s5-\mathrm{sim}}$/$\phi_{s10-\mathrm{sim}}$, respectively.

## 5. Implications

### 5.1. Implications for the Characterization of Pore Structure of Porous Materials by MIP Measurement

According to Hill (1963) [38], in the theory of composite materials, the representative elementary volume (REV) is the smallest volume over which a measurement can be made that will yield a value representative of the whole. This study simulated mercury intrusion into artificial sandstone and proved that the effect of sample size (see Figure 12) always exists before the mercury-intruded pores are percolated in the sandstone samples. This indicates that there is no REV for MIP measurements on the artificial sandstone investigated in this study. 

**Figure 13:**
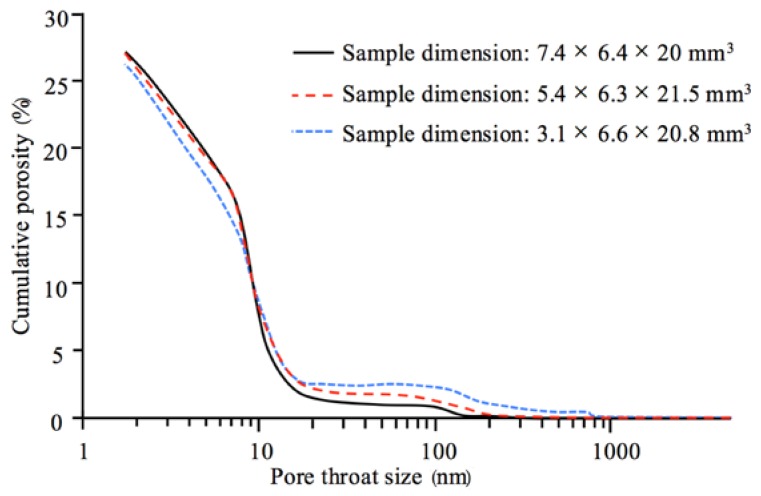
Comparison of the pore throat size distribution (PSD) curves of cement pastes (water-to-cement ratio = 0.5, cured in water for 2 years) with different dimensions. The PSD curves were determined by MIP measurement [17].

As mentioned in Section 1, the effect of sample size on mercury intrusion into cement-based materials has been reported [17,20,22,23]. The breakthrough pore size of cement-based materials may range from nm to μm, depending on water-to-cement ratio and curing time [39]. Figure 13 presents the PSD curves of cement pastes (water-to-cement ratio = 0.5, cured in water for 2 years) with different dimensions (data derived from [17]). The breakthrough pore size of the cement pastes appears to be between 10–20 nm. Smaller samples showed a higher cumulative porosity at low pressures (or large pore throat sizes; e.g., $d_{c}$ > 20 nm). The effect of sample size of the cement paste on the measured cumulative porosity is in good agreement with the work presented in this study. Therefore, in general, the effect of sample size needs to be considered in MIP measurements on porous materials. A fixed sample size should be used in MIP measurements for comparison of the pore structure between different materials. 

### 5.2. Implications for the Characterization of the Pore Structure of Porous Materials by MIP Simulation

**Figure 14:**
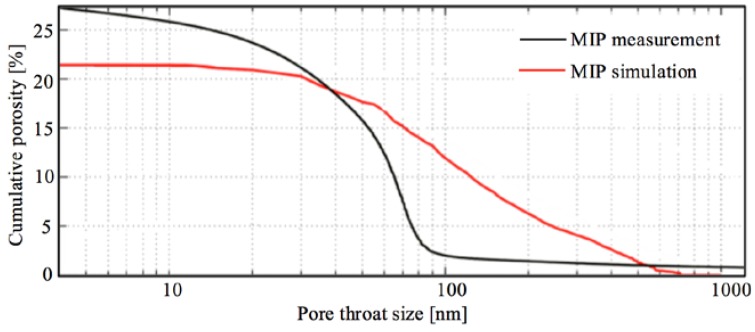
Comparison of PSD curves of a cement paste (OPC CEM I 42.5, water-to-cement ratio (w/c) 0.35, after 28 days of hydration) determined by MIP measurement and MIP simulation [25].

In the literature, MIP simulation has been conducted to determine the PSD curves of digital microstructures of cement-based materials [25,26]. The PSD curves are usually compared with those determined by MIP measurements. Noticeable discrepancy in the PSD curves was observed. Figure 14 gives an example of the comparison of PSD curves of a cement paste (OPC CEM I 42.5, w/c 0.35, after 28 days of hydration) that were determined by MIP measurement and MIP simulation, respectively [25]. The digital microstructure of the cement paste used for MIP simulation was obtained using Focused ion beam-nanotomography. The digital microstructure had a voxel size of 14.84 nm × 18.84 nm × 30.0 nm and a voxel number of 800 × 570 × 185, yielding a data volume of 11.9 μm × 10.7 μm × 5.6 μm. The PSD curve determined by MIP simulation indicates a much coarser pore structure than that indicated by the PSD curve determined by MIP measurement. Note that the size of the digital microstructure of the cement paste (i.e., 11.9 μm × 10.7 μm × 5.6 μm) is 2–3 orders of magnitude smaller than that used in MIP measurements (e.g., several millimeters). According to the study presented in Section 4, the effect of sample size is believed to be one of the main reasons for the discrepancy in the PSD curves observed in Figure 14. However, the effect of sample size on the determined PSD curves is commonly overlooked. For a valid comparison of the PSD curves of porous materials, the same sample size should be used in MIP simulations and MIP measurements.

### 5.3. Implications for the Application of Pore Throat Size in the Estimation of Air Permeability

Natural rocks have a wide range of pore throat sizes, from nm to μm [27,40]. The pore throat sizes of natural rocks determined by MIP have been used to estimate their air permeability, while the sample size used for MIP measurements was often not reported [14,15]. Based on the findings in relation to the effect of the sample size of artificial sandstone on the determined pore throat size, implications are given for the application of the pore throat size in the estimation of air permeability.

Pittman (1992) [14] analyzed the air permeability, the porosity, and the pore throat sizes of 202 sandstone samples. The porosities and the air permeabilities of the 202 sandstone samples ranged from 3.3 to 28% and 0.05 to 998 mD, respectively. It was found that the use of a pore throat size corresponding to the 25th percentile of mercury saturation gave the best correlation (Equation (4)) [14].

(4) $$\mathrm{Log}\;K=-1.221+1.415\;\mathrm{Log}\;\phi +1.512{\;\mathrm{Log}\;r}_{25}$$

where K is the uncorrected air permeability (mD), ϕ is porosity (%), and $r_{25}$ is the pore throat radius corresponding to the 25th percentile of the saturation of mercury on a cumulative mercury injection plot (analogous to the PSD curves determined by using MIP technique).

**Figure 15:**
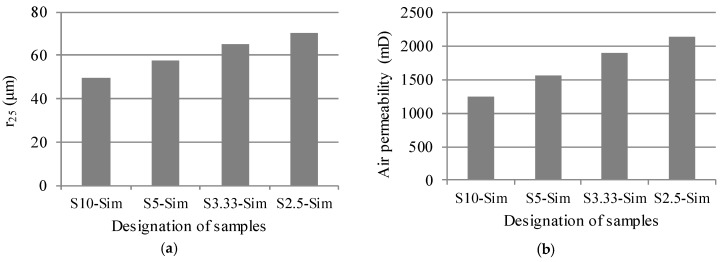
Calculation of air permeability of the samples with different sizes. (**a**) Pore throat radius corresponding to the 25th percentile of saturation of mercury. (**b**) Calculated permeability of the samples. The air permeability was calculated from the porosity and the pore throat radius according to Equation (4).

The significance of sample size of sandstone in the calculation of air permeability with Equation (4) is investigated in this study. The pore throat radii corresponding to the 25th percentile of saturation of mercury for artificial sandstone samples with different sizes were determined from the PSD curves (see Figure 10a) and shown in Figure 15a. The value of $r_{25}$ increased with the decrease of sample size. The pore throat size corresponding to $r_{25}$ was 99 μm, 115 μm, 130 μm, and 141μm for S10-Sim, S5-Sim, S3.33-Sim, and S2.5-Sim, respectively. At these pore throat sizes, mercury still had not percolated through the samples (see Figure 11), and the sample size had a significant effect on the volume of mercury-intruded pores (see Figure 10a and Figure 12).

Air permeability of the artificial sandstone was calculated with Equation (4) and shown in Figure 15b. The calculated air permeability of the smaller sandstone sample was higher (e.g., the calculated air permeability of S2.5-Sim was 1.7 times higher than that of S10-Sim). However, in principle, the air permeability of sandstone samples should be independent of the sample size. This indicates that Equation (4) is conditional on a given sample size. This case study suggests that the sample size needs to be taken into account for a proper use of pore throat size of porous materials. 

## 6. Conclusions

This paper investigated the effect of sample size on mercury intrusion into an artificial sandstone using MIP measurements and MIP simulations. The digital microstructures of sandstone used in MIP simulation were reconstructed from CT scan data. Percolation analysis of mercury-intruded pores was conducted to evaluate the effect of the sample size of digital microstructures. The pore throat size of artificial sandstone was used to estimate their air permeability using the relation proposed in the literature. The following conclusions can be drawn:

- For a cubic sample with a length 1/*n* times that of the reference cubic sample, the mercury-intruded porosity of the small sample was scaled up by a factor of approximately n under low pressure. The effect of sample size decreased with increasing pressure and became minor after mercury-intruded pores percolated through the samples. It can be concluded that there was no representative elementary volume (REV) in MIP measurements or MIP simulations for the sandstone samples investigated in this study, because a significant effect of sample size always existed before the mercury-intruded pores were percolated in the samples. For a proper comparison of pore structure between the artificial sandstone, the same sample size should be used in MIP measurement and MIP simulation.
- The pore throat radius ($r_{25}$) corresponding to the 25th percentile of saturation on a cumulative mercury injection plot was used to estimate the air permeability of artificial sandstone, according to the relation proposed in the literature. The calculated air permeability of the smaller sandstone sample had higher air permeability (the calculated air permeability of S2.5-Sim was 1.7 times higher than that of S10-Sim). However, in principle, the air permeability of sandstone samples should be independent of the sample size. This suggests that sample size needs to be considered when pore throat size determined by MIP measurement is used for estimating the air permeability of rocks.

## Figures and Tables

**Table 1:** Samples for mercury intrusion porosimetry (MIP) measurements, X-ray computed tomography (CT) scan test, and MIP simulation.

Designation	Dimension (mm)	Scale Factor n	Number of Samples	Description
S10-Exp	10×10×10	1	2	Samples for MIP test ^1^
S5-Exp	5×5×5	2	8	
C15	Diameter = 15, height = 15	-	1	Samples for CT scan test ^2^
S10-Sim	10×10×10	1	1	Samples for MIP simulation ^3^
S5-Sim	5×5×5	2	8	
S3.33-Sim	3.33×3.33×3.33	3	27	
S2.5-Sim	2.5×2.5×2.5	4	64	

^1^ The samples were cut from the bulk specimen (size: $40\times 40\times 40\;{\mathrm{mm}}^{3}$). The 5 mm-thick surface layers of the bulk specimen were disposed of before preparing S10-Exp and S5-Exp; ^2^ The sample was cut from the bulk specimen (diameter = 15 mm, height = 100 mm). The 5 mm-thick top and bottom surface layers of the bulk specimen were disposed of before preparing C15; ^3^ Digital microstructures of the samples were extracted from that of C15.

**Table 2:** Description of digital samples for MIP simulation. GSV: greyscale value.

Designation	S10-Sim	S5-Sim	S3.33-Sim	S2.5-Sim
**Physical Dimension (mm^3^)**	10×10×10	5×5×5	3.33×3.33×3.33	2.5×2.5×2.5
**Digital resolution**	10 μm/voxel			
**Digital dimension (voxel^3^)**	1000×1000×1000	500×500×500	333×333×333	250×250×250
**Segmentation method**	Voxels are identified as pores when 0≤GSV ≤49, epoxy resin when 49<GSV ≤111 and quartz sand when 111<GSV ≤255.			
**Number of samples**	1	8	27	64
**Average porosity**	17.44%	17.44% ± 0.66%	17.44% ± 1.01%	17.44% ± 1.15%
**Epoxy resin (V%)**	18.6%	18.6% ± 0.4%	18.6% ± 0.63%	18.6% ± 0.76%
**Quartz sand (V%)**	64.2%	64.2% ± 0.3%	64.2% ± 0.59%	64.2% ± 0.73%
